# Protein bodies: how the ER deals with high accumulation of recombinant proteins

**DOI:** 10.1111/pbi.12730

**Published:** 2017-05-10

**Authors:** Reza Saberianfar, Rima Menassa

**Affiliations:** ^1^ London Research and Development CentreAgriculture and Agri‐Food CanadaLondonONCanada; ^2^ Biology DepartmentUniversity of Western OntarioLondonONCanada

**Keywords:** protein body, endoplasmic reticulum, fusion tags, elastin‐like polypeptide, hydrophobin, Zera®

Protein bodies (PBs) are highly specialized protein storage organelles in cereal seeds. PB formation in seeds initiates in the endoplasmic reticulum (ER), and depending on the plant species, PBs remain in the ER or find their way out of the ER, bypass the Golgi and end up in protein storage vacuoles (PSVs) (Khan *et al*., 2012).

Protein bodies have been ectopically induced in leaves by producing high amounts of ER‐retrieved recombinant proteins, usually by fusing them to protein tags such as Zera®, elastin‐like polypeptide (ELP) and hydrophobin‐I (HFBI). PBs induced by these processes are numerous, round and clustered, they remain in the ER, and they are not destined to the central vacuole (Llop‐Tous *et al*., 2010; Saberianfar *et al*., 2015, 2016).

## PB formation initiates upon reaching a threshold level

**Figure 1:**
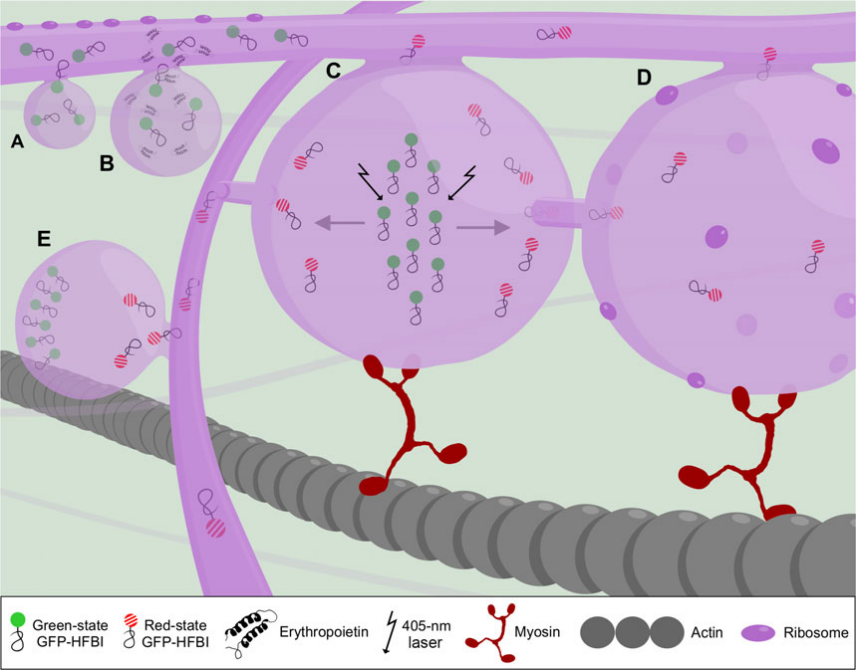
A working model of protein body formation and development. (A) Proteins are synthesized on the rough endoplasmic reticulum (ER) by ribosomes and transferred into the ER lumen co‐translationally. Protein body formation initiates when localized high concentrations of recombinant proteins occur in the ER lumen. High concentration of proteins is represented by several GFP‐HFBI molecules in an area (GFP‐HFBI is presented here as an example. These properties can be generalized to other protein fusions as well). (B) Once protein bodies (PBs) form, they grow in size over time and store higher amounts of proteins in their lumen. Co‐expression of high‐value recombinant proteins, in this case erythropoietin (EPO), with GFP‐HFBI results in passive sequestration of EPO molecules into GFP‐HFBI‐induced PBs. (C). PBs remain connected with the ER and exchange their content with other PBs via the ER or by direct contact. (D) PBs are part of the rough ER and studded with ribosomes. These ribosomes may contribute to accumulation of proteins in PBs. (E) The ER network is essential in connecting PBs that are located far from each other. In this model, GFP‐HFBI photoconverts to red‐state GFP‐HFBI upon irradiation (shown with arrows) (C). Photoconverted proteins move from one PB to neighbouring PBs (D) and to far away PBs through the ER (E). The ER and PB movement relies on the actomyosin cytoskeleton.

The simultaneous monitoring of recombinant protein accumulation and PB formation suggested that a recombinant protein threshold of 0.2% total soluble protein is required for PB formation (Figure 1A). This observation was based both on expression of fluorescent proteins in transgenic tobacco lines (Gutiérrez *et al*., 2013), and on transient expression of fluorescent proteins as well as nonfluorescent xylanases in *Nicotiana benthamiana* (Saberianfar *et al*., 2015).

Although fusion tags are not necessary for PB formation, their presence affects the distribution pattern and size of PBs (Saberianfar *et al*., 2015). This can be due to their physicochemical properties. Indeed, all three types of fusion tags possess hydrophobic regions, which enable fusion tags and their fusion partners to self‐assemble into aggregates and facilitate the process of PB formation.

Once PBs arise, they grow in size over time (Figure 1B), reaching their maximum size at 5 days post infiltration (dpi) for GFP‐ELP and GFP‐HFBI (Saberianfar *et al*., 2015), and at 10 dpi for Zera®‐ECFP (Llop‐Tous *et al*., 2010). In all cases, PB growth is accompanied by an increase in accumulation levels of the respective recombinant protein.

## Proteins are sequestered passively into some, but not all, PBs

Once PBs start forming, they can trap endogenous and other co‐expressed proteins. Indeed, PBs were shown to contain ER‐specific proteins such as BiP, calreticulin and calnexin (Conley *et al*., 2009; Joseph *et al*., 2012). As a result, other proteins of interest can be trapped into PBs when co‐expressed with PB‐inducing proteins. For instance, GFP targeted either for secretion or for retrieval to the ER was sequestered into PBs upon co‐expression with RFP‐HFBI or RFP‐ELP. Also, co‐expression of valuable and low‐accumulating proteins such as erythropoietin and interleukin‐10 with GFP‐HFBI led to increased accumulation of both proteins (Saberianfar *et al*., 2015). Such proteins are likely sequestered in PBs where they are prevented from progressing through the secretory pathway, and over time, their concentration in PBs increases (Figure 1B). This PB‐trapping strategy is important for two reasons; it helps to increase accumulation levels of difficult‐to‐express recombinant proteins and eliminates the need for the addition of fusion tags which might affect the proper folding and activity of the protein of interest.

This strategy, however, does not work as well with Zera®, because the strong affinity of Zera® molecules to one another results in a condensed and sticklike alignment of Zera® molecules which excludes other molecules from the core of PBs (Llop‐Tous *et al*., 2010). This was confirmed with co‐expression of secretory GFP or ER‐targeted GFP with Zera®‐DsRed in which the GFP signal localized to the periphery of Zera® PBs and not in their core, and explains why Zera®‐DsRed has no significant effect on EPO accumulation (Saberianfar *et al*., 2016).

## ER is the initiation point and the final destination of PBs

Previous studies on PBs have interpreted the subcellular localization of PBs differently. Zera®‐induced PBs were described as highly dense aggregates, which remain connected to the lumen of the ER rather than forming independent organelles (Llop‐Tous *et al*., 2010). Alternatively, ELP and HFBI PBs were described as terminally stored organelles, which bud off the ER and are highly mobile in the cytosol (Joensuu *et al*., 2010).

We recently showed that physical connections exist between PBs induced by ELP, HFBI and Zera® using a new GFP photoconversion technique (Sattarzadeh *et al*., 2015). Confocal imaging of connections between PBs using fluorescent proteins is technically challenging. PBs induced by fluorescent proteins generally appear extremely bright under the confocal microscope due to the accumulation of high levels of fluorescent proteins in their lumen. Therefore, high‐quality imaging is only possible under excitation with low laser power and minimal gain. In these conditions, the ER network connecting PBs, which contains low amounts of the fluorescent proteins, is not visible. Due to these technical difficulties, photoconversion is an important tool to monitor the movement of proteins between PBs and allows imaging of protein trafficking out of and into PBs.

Based on our observations, in the case of ELP and HFBI, photoconverted proteins within PBs were rapidly transferred, first to surrounding PBs (Figure 1C,D), and then to distant PBs within the cell via the ER (Figure 1E). In the case of Zera®, protein movement between PBs was also observed, but was much slower, likely due to covalent disulphide bridges between Zera® molecules leading to large aggregates (Saberianfar *et al*., 2016). These results indicate that PBs are linked to each other through the ER and that PBs are not terminally stored cytosolic organelles. Another technique to show the connection of PBs is transmission electron microscopy (TEM). For a TEM image to capture PB connections, it is critical that the specimen be sectioned at a focal plane that shows these connections similar to what was shown by Hofbauer *et al*. (2014). As sample preparation is a random process, in many cases PB connections are not visible. The ideal technique to capture the details of PB connections would be electron tomography that can provide 3D images based on multiple sections of a cluster of PBs.

In higher plants, ER formation and movement are actin‐dependent via interaction of actin microfilaments with myosin XI proteins. The movement of PBs induced by Zera®, ELP and HFBI fusion tags was disrupted by the use of Latrunculin B, an actin depolymerizing drug, and ELP‐induced PBs were shown to lose mobility in the presence of a mutant myosin XI‐K tail. Furthermore, PBs form and align along actin strands and are surrounded by an ER membrane (Conley *et al*., 2009; Saberianfar *et al*., 2016). Therefore, we speculate that PBs remain connected to the ER, and move along the ER network which relies on the actin cytoskeleton for movement (Figure 1).

## Future prospects

It is not fully clear whether high accumulation of proteins causes the formation of PBs or whether the formation of PBs is responsible for accumulation of high amounts of recombinant proteins. We believe reaching a certain protein accumulation in the ER is critical in PB initiation, and other factors such as presence or absence of the fusion tags contribute to PB maturation. Understanding the mechanism of PB formation will help us use this technology for increasing the yield of foreign proteins in plant expression systems.

The differences between the fusion tags and how they direct their fused partner to PBs need further investigation. We showed that all three types of PBs arise from the ER, but that Zera®‐induced PBs do not co‐localize with either ELP‐ or HFBI‐induced PBs. Unlike ELP and HFBI, Zera® does not require an ER retrieval signal to accumulate in the ER and form PBs. It is possible that Zera®‐induced PBs originate from a different subdomain of the ER compared to ELP‐ or HFBI‐induced PBs. This can be examined for instance by co‐expression with ER subdomain‐specific proteins. Although PBs induced by fusion tags show differences, this feature might be useful for simultaneous expression of different proteins *in vivo* and their targeting into the same or separate PBs, especially as Zera®, ELP and HFBI can be isolated and purified by different purification strategies.

Even though it is clear that all fusion‐induced PBs exchange their contents through the ER, we cannot rule out the potential role of *de novo* protein synthesis by ribosomes attached to the rough ER membrane of Zera®‐, ELP‐ and HFBI‐induced PBs. This question can be addressed by a photoconversion‐based FRAP experiment in the presence and absence of an inhibitor of protein synthesis (e.g. cycloheximide), in which fluorescence recovery is measured.

In conclusion, induction of PB formation enables the storage of high amounts of intracellular recombinant proteins without imposing excessive stress to the ER, and may be a coping mechanism that eukaryotic cells have evolved to prevent ER stress and cell death. We believe that PBs work as extensions of the ER and provide sufficient space for protein accumulation. Taking advantage of this phenomenon can constitute an approach to addressing the production bottleneck of low recombinant protein accumulation levels in plants, especially in leaf‐based expression systems.
